# Annexin A2 facilitates porcine circovirus type 2 infection by mediating viral attachment to host cells and interacting with the capsid protein

**DOI:** 10.1186/s13567-025-01628-3

**Published:** 2025-10-10

**Authors:** Yifan Jiang, Xinnuo Lei, Weijiao Liu, Siyu Cao, Qing He, Xiaohong Xie, Yang Zhan, Lei Tan, Jinhui Mai, Lingchen Yang, Aibing Wang, Yi Yang, Naidong Wang

**Affiliations:** 1 Hunan Provincial Key Laboratory of Protein Engineering in Animal Vaccines, Laboratory of Functional Proteomics (LFP) & Research Center of Reverse Vaccinology (RCRV), College of Veterinary Medicine, Hunan Agricultural University, Changsha, China; 2 Jiangsu Key Laboratory for High-Tech Research and Development of Veterinary Biopharmaceuticals, Engineering Technology Research Center for Modern Animal Science and Novel Veterinary Pharmaceutical Development, Jiangsu Agri-Animal Husbandry Vocational College, Taizhou, China; 3 College of Animal Science and Technology, Yangtze University, Jingzhou, China

**Keywords:** Porcine circovirus type 2, capsid, ANXA2, cell binding

## Abstract

Porcine circovirus type 2 (PCV2) causes substantial economic losses globally. Although glycosaminoglycans (GAGs) have been identified as the general cell receptors responsible for PCV2 binding and subsequent cell infection, other critical protein(s) may also be involved. Our objective was to explore key host proteins that mediate PCV2 infection. The host protein annexin A2 (ANXA2) was observed to interact with PCV2 virus-like particles (VLPs) through immunoprecipitation (IP) and liquid chromatography‒tandem mass spectrometry (LC‒MS/MS) experiments. Furthermore, knockdown of ANXA2 or the inhibitor A2ti-1 significantly reduced PCV2 replication in host cells. Notably, the addition of ANXA2-specific antibodies to cell culture or the preincubation of PCV2 with recombinant ANXA2 significantly reduced viral infection, primarily by weakening viral attachment to cells. Confocal microscopy further confirmed the colocalization of PCV2 Cap with endogenous ANXA2 in infected cells, and in vitro GST pull-down assays also demonstrated a direct interaction between these two proteins, confirming the role of ANXA2 as an attachment factor for PCV2 cellular entry. In addition, molecular docking revealed that an interface and multiple residues between PCV2 Cap and ANXA2 were involved in the interaction, which was also verified by truncation assays. In conclusion, ANXA2 on the cell surface may function as an attachment site, promoting viral binding and increasing PCV2 infectivity through interactions with Cap. These findings provide novel insights into the molecular mechanisms of PCV2 infection and new molecular targets for the development of vaccines and therapeutic strategies.

**Supplementary Information:**

The online version contains supplementary material available at 10.1186/s13567-025-01628-3.

## Introduction

Porcine circovirus (PCV), a member of the *Circoviridae* family, is a nonenveloped, single-stranded circular DNA virus with an icosahedral morphology [1]. There are four PCV genotypes (PCV1–4), and the PCV3 genome is approximately 2.0 kb in length, whereas the PCV1, PCV2, and PCV4 genomes are approximately 1.7 kb in length [2–4]. Although PCV2 primarily targets porcine monocytes/macrophages, it can also infect and replicate within epithelial cells. It is the main aetiological agent of porcine circovirus-associated diseases (PCVADs) and plays an important role in immunosuppression and is frequently associated with coinfections of other pathogens in swine herds, thereby accelerating disease progression. The PCV2 genome has 1767 or 1768 bases, containing 11 open reading frames (ORFs), with ORF1 and ORF2 encoding two major viral proteins [5]; ORF1 encodes Rep and Rep’, both of which are essential for virus replication [6, 7], whereas ORF2 encodes capsid protein (Cap), the sole structural protein of PCV2 [8]. Cap, the primary constituent of the PCV2 capsid with a typical jelly roll fold, plays a key role in the PCV2 life cycle, including mediating viral attachment to cells and the subsequent internalization of viruses through host cell receptors [9, 10]. In vitro, 60 PCV2 Cap can self-assemble into a virus-like particle (VLP) that structurally resembles the wild-type virus and is capable of binding and invading host cells [11]. VLPs are valuable tools for studying molecular mechanisms involved in viral attachment and cellular entry.

Understanding initial viral attachment and cellular entry mechanisms is essential for the development of effective antiviral strategies, as this stage represents an ideal time frame to prevent infection. Heparan sulfate (HS) and chondroitin sulfate B (CS-B), both of which are glycosaminoglycans (GAGs), are the primary functional receptors for PCV2 attachment [10]. In monocytes and epithelial cells, the PCV2 capsid primarily attaches to the cell surface by binding to these GAGs, initiating subsequent cell infection [10, 12]. Additionally, Cap can interact with other cell surface molecules, such as P-selectin and phosphacan, the latter being a GAG-containing protein expressed on porcine peripheral blood monocytes, to facilitate viral attachment and internalization [13, 14]. Although these findings emphasize the role of host molecules in PCV2 infection, capsid-mediating host molecules involved in the adsorption and infection processes of PCV2 are not well characterized.

Annexin A2 (ANXA2), a calcium-dependent phospholipid-binding protein, is strategically located on the cell membrane and has a strong affinity for HS chains [15]. ANXA2 plays a key role in viral entry and the adhesion of pathogenic microorganisms to host cells [16, 17]. For example, cell surface ANXA2 is critical for the internalization of human papillomavirus (HPV) and is also a potential receptor for enterovirus 71 (EV71) through interactions with the capsid protein VP1 [18, 19]. During avian reovirus (ARV) infection, cell surface ANXA2 collaborates with ADGRL2 to increase ARV entry [20]. Furthermore, the C-terminal domain of ANXA2 binds F-actin and heparan sulfate proteoglycans (HSPGs), facilitating pathogen internalization and cellular uptake, as demonstrated by its role in mediating the uptake of extracellular vesicles via HS [16, 17]. Notably, ANXA2 also mediates the adherence of *Streptococcus anginosus* through interactions with its surface protein TMPC, contributing to tumorigenesis [21]. Despite its importance in various infections, the role of ANXA2 in PCV2 infection is unknown.

Our aims were to identify key host protein(s) involved in PCV2 adsorption and infection and to further investigate their functions associated with viral infection. For this purpose, PK-15 cells were incubated with PCV2 VLPs, after which coimmunoprecipitation (Co-IP) and liquid chromatography‒tandem mass spectrometry (LC‒MS/MS) were performed. Consequently, ANXA2 was identified as a novel host protein capable of interacting with PCV2 Cap during the initial stage of PCV2 infection, as demonstrated by antibody blockage and competitive binding assays. These findings contribute to a deeper understanding of the molecular mechanisms underlying PCV2 infection and reveal a novel potential target for vaccine development and therapeutics against PCV2 infection.

## Materials and methods

### Cells, viruses and VLPs

Porcine kidney (PK-15) and 293FT cells were grown in complete Dulbecco’s modified Eagle’s medium (DMEM; Invitrogen, Carlsbad, CA, USA) supplemented with 10% (v/v) heat-inactivated foetal bovine serum (FBS; Atlanta Biologicals, Minneapolis, MN, USA) and 1% penicillin‒streptomycin (Gibco, Waltham, MA, USA). The cells were cultured at 37 °C in a 5% CO_2_ incubator. The PCV2 genotype 2d strain ChenZ-2-1 (GenBank accession number: MH718995), stored in our laboratory, was the genetic source for the PCV2 VLPs used in this study. PCV2 VLPs were assembled from the Cap, expressed and purified as described previously [11].

### Plasmid construction and transfection

The pCI-neo-Cap/flag plasmid was stored in our laboratory. The cDNA of porcine full-length ANXA2 (NCBI reference sequence: NM_001005726.1) was amplified from porcine lung tissues and subcloned and inserted into the pCDNA3.4 vector, with an HA tag fused to its C-terminal end. To identify the ANXA2 domains responsible for the interaction with Cap, six truncated ANXA2 mutants were designed, synthesized and subcloned and inserted into the pCDNA3.4 vector, each containing a C-terminal HA tag, for coimmunoprecipitation assays. A schematic representation and the amino acid ranges of each mutant are shown in Figure 7A. Two specific short hairpin RNAs (shRNAs) targeting ANXA2 were designed using the BLOCK-iT™ RNAi Designer online tool [22]. The sequences of the shRNAs targeting porcine ANXA2 were as follows: shANXA2-1 (sense 5’-3’: CCGGGCCTTTGCCTACCAAAGAAGGTCAAGAGCCTTCTTTGGTAGGCAAAGGCTTTTT; antisense 5’-3’: AATTAAAAAGCCTTTGCCTACCAAAGAAGGCTCTTGACCTTCTTTGGTAGGCAAAGGC), shANXA2-2 (sense 5’-3’: CCGGGCGTGATAAGGTCCTGATTAGTCAAGAGCTAATCAGGACCTTATCACGCTTTTT; antisense 5’-3’: AATTAAAAAGCGTGATAAGGTCCTGATTAGCTCTTGACTAATCAGGACCTTATCACGC). Concurrently, sequences targeting non-ANXA2 regions were designed as controls and designated shNC. All the designed DNA sequences were synthesized by Tsingke (Beijing, China) and subsequently cloned and inserted into the pLKO.1-EGFP-Puro vector. PK-15 cells were transfected with plasmids expressing the respective shRNAs for 24 h, followed by infection with PCV2 for an additional 36 h. Whole-cell lysates were collected for western blot analysis, and PCV2 DNA was extracted from both cells and the supernatant to determine viral genome copy numbers.

### Recombinant protein expression and purification

Full-length porcine ANXA2 and truncated ANXA2-M1 (1–102 aa), originally constructed for mammalian expression, were subcloned and inserted into the bacterial expression vector pET-28a (+) (Novagen). A deletion mutant lacking repeat II (122–174 aa) (designated ANXA2-△II) was synthesized and cloned directly into the pET-28a (+) vector by Sangon Biotech (Shanghai, China). All the constructs contained an N-terminal His-tag for purification.

The recombinant proteins were expressed in *Escherichia coli* BL21(DE3) cells. Cultures were grown at 37 °C to an optical density at 600 nm (OD600) of approximately 0.6, and protein expression was induced with 1 mM isopropyl β-D-1-thiogalactopyranoside (IPTG) at 26 °C for 6 h. The cells were subsequently harvested and centrifuged at 12 000 × *g* for 20 min at 4 °C. Soluble proteins were purified as previously described [11]. Purified protein concentrations were determined using a BCA protein assay kit (Thermo Fisher Scientific).

### Immunoprecipitation and mass spectrometry

PK-15 cells were seeded in 10-cm cell culture dishes at a density of 5.0 × 10^6^ cells/dish and then mixed with PCV2 VLPs for 2 h, after which the cells were washed three times with cold PBS and lysed with WB/IP buffer (no. R0100; Solarbio, Beijing, China) supplemented with a protease inhibitor cocktail (no. B14001; Selleckchem, Houston, TX, USA). After centrifugation (10 000 × *g*, 5 min), the supernatant was collected and incubated with Dynabeads Protein G (no. 1003D; Invitrogen) that had been preincubated with anti-PCV2 Cap antibody (stored in our laboratory) at 4 °C for 1 h with rotation. Immune complexes were washed three times with PBST (with 0.02% Tween-20) and eluted with 50 mM glycine (pH 2.8). Immunoprecipitants were subjected to sodium dodecylsulfate-polyacrylamide gel electrophoresis (SDS‒PAGE), and the gel was excised for LC‒MS/MS.

### Western blot

Cells were lysed in RIPA buffer (no. R0010; Solarbio) supplemented with protease and phosphatase inhibitors (Selleckchem) for 30 min on ice. Protein concentrations were determined using a BCA Protein Assay Kit (no. 23227; Thermo Fisher Scientific, MA, USA). Equal amounts of protein were mixed with 5 × loading buffer (containing 0.05% β-mercaptoethanol) and boiled for 5 min. The proteins were separated by SDS‒PAGE before being transferred to a 0.22-μm polyvinylidene difluoride (PVDF) membrane (Millipore, Temecula, CA, USA). Thereafter, the membrane was blocked with 5% nonfat dry milk and incubated with the following primary antibodies: anti-PCV2 Cap (stored in our laboratory), anti-HA (no. 66006-2-Ig, Proteintech, Wuhan, China), anti-ANXA2 (no. 11256-1-AP, Proteintech), or anti-β-actin (no. 81115–1-RR, Proteintech) and corresponding horseradish peroxidase (HRP)-conjugated goat anti-rabbit IgG (H + L) (no. ab205718, Abcam, USA) or HRP-conjugated goat anti-mouse IgG (H + L) secondary antibodies (no. ab205719, Abcam). The protein bands were visualized using Immobilon Forte Western HRP Substrate (no. WBLUF0100; Millipore) and captured on a chemiluminescence imager (Bio-Rad; Hercules, CA, USA).

### Immunofluorescence assay and confocal microscopy

PK-15 cells were seeded on cover slips in a 12-well plate at a density of 2.0 × 10^5^ cells/well. After adhering, the cells were infected with PCV2 (1 MOI) and incubated at 37 °C for the indicated time. Following infection, the cells were washed three times with PBS, fixed with 4% paraformaldehyde (Solarbio) for 20 min at room temperature, permeabilized with 0.1% Triton X-100 (Solarbio) for 10 min, and blocked with 3% BSA for 1 h. Next, the cells were incubated with mouse anti-PCV2 Cap antibody (stored in our laboratory) and rabbit anti-ANXA2 monoclonal antibody (no. ET1704-49; HUABIO, Hangzhou, China) overnight at 4 °C, followed by further probing with Alexa Fluor 594-conjugated donkey anti-rabbit IgG (no. A-21207; Invitrogen) and Alexa Fluor 488-conjugated donkey anti-mouse IgG (no. A-21202; Invitrogen) for 1 h at 37 °C. Nuclei were stained with ProLong® Gold anti-fade reagent with 4',6-diamidino-2-phenylindole (DAPI) (no. P36935; Invitrogen). Images were visualized under Leica Application Suite Advanced Fluorescence LAS AF v. 2.2.1 (Leica Microsystems CMS GmbH, Germany). To quantify the colocalization of ANXA2 and Cap, Pearson’s correlation coefficient (PCC) was calculated using ImageJ/Fiji software. The analysis was conducted on three independent experiments, and a PCC > 0.5 indicated strong colocalization.

To detect plasma membranes and identify surface ANXA2, PK-15 cells were stained with CellMask™ Green Plasma Membrane Marker (no. C37608; Invitrogen) for 20 min, fixed, and incubated with anti-ANXA2 monoclonal antibody (no. ET1704-49; HUABIO) at 4 °C for 30 min under nonpermeabilized conditions. The cells were subsequently incubated with Alexa Fluor 594-conjugated donkey anti-rabbit IgG (no. A-21207; Invitrogen) at 4 °C for 30 min. Images were acquired as described above.

### Inhibitor treatments

PK-15 cells were pretreated with dimethyl sulfoxide (DMSO, 0.2%, V/V, no. 276855; Sigma‒Aldrich, St. Louis, MO, USA) or A2ti-1 (no. HY-136465; MedChemExpress, Monmouth Junction, NJ, USA) at 37 °C for 2 h, infected with PCV2 for 1 h, and finally cultured for an additional 36 h in fresh medium supplemented with inhibitor.

### RT‒qPCR and qPCR analysis

Total RNA was isolated using TRIzol reagent (no. R1100; Solarbio) and reverse transcribed into complementary DNA (cDNA) using Moloney Murine Leukaemia Virus Reverse Transcriptase (M-MLV RT) (no. M1701; Promega Biotech Co., Ltd., Beijing, China) according to the manufacturer’s instructions. Quantitative real-time PCR (RT‒qPCR) was subsequently performed using ChamQ Universal SYBR qPCR Master Mix (no. Q711-02; Vazyme Biotech Co., Ltd., Nanjing, China) in a QuantStudio™ Real-Time PCR System (Thermo Fisher Scientific). Relative mRNA levels were quantified by normalization to those of the housekeeping gene TBP (TATA-box binding protein) and calculated using the 2^−ΔΔCT^ method. We used the following primers: porcine TBP, forwards, 5’-GATGGACGTTCGGTTTAGG-3’ and reverse, 5’-AGCAGCACAGTACGAGCAA-3’ [23]; and porcine ANXA2, forwards, 5’-GGAGTGTGTGTCACCTCCAG-3’ and reverse, 5’-AGTTGTACAGGGACTTGCCG-3’. DNA from cells was extracted using a TIANamp Genomic DNA Kit (no. DP304; TIANGEN, Beijing, China), whereas PCV2 viral DNA from the supernatant was isolated using an AxyPrep Body Fluid Viral DNA/RNA Miniprep Kit (no. AP-MN-BF-VNA-250; Axygen Scientific, USA). Quantitative PCR (qPCR) amplification and quantification of PCV2 copy numbers were performed as previously described [24].

### Flow cytometry

For each assay, PK-15 cells were seeded in 6-well cell plates at a density of 1.0 × 10^6^ cells/well and then were washed three times with staining buffer (no. 554656; BD Pharmingen, USA) and blocked with 3% BSA in staining buffer for 30 min. The cells were then incubated with either anti-ANXA2 monoclonal antibody (20 μg/mL; HUABIO) or a rabbit IgG isotype control antibody (no. HA722127; HUABIO) at 4 °C for 30 min. After being washed and centrifuged (300 × *g* for 1 min), the cells were resuspended in staining buffer and incubated with Alexa Fluor 488-conjugated donkey anti-rabbit IgG secondary antibody for 30 min at 4 °C; the data were analysed using a BD Accuri C6 Plus flow cytometer, and the data were processed with FlowJo software (version 10.8.1).

### Antibody blocking assay

PK-15 cells were cultured as a monolayer at 37 °C at a density of 1.0 × 10^5^ cells/well and then preincubated with specific rabbit anti-ANXA2 monoclonal antibodies targeting the N-terminal region (amino acids 26–75) (no. ET1704-49; HUABIO) and a rabbit anti-ANXA2 polyclonal antibody (no. HA722127; Proteintech) at final concentrations of 10 and 30 μg/mL, respectively, for 1 h. Additionally, an isotype control antibody was used at a final concentration of 30 μg/mL. Cells were subsequently infected with PCV2 for 1 h. After three washes with PBS, the cells were incubated at 37 °C for 36 h. Finally, cellular DNA was extracted, and viral copy numbers of PCV2 were determined using qPCR.

### Recombinant soluble ANXA2 competition assay

Prior to infecting PK-15 cells, PCV2 was preincubated with varying concentrations (2, 5 or 20 μg/mL) of recombinant soluble ANXA2 (rANXA2) or BSA at 37 °C for 1 h. Subsequently, the virus was allowed to infect the cells for 1 h. After three washes with PBS, the cells were incubated at 37 °C for 36 h. Cellular DNA was extracted, and viral copy numbers were assessed using qPCR.

### Virus binding assay

For the antibody blocking assay, PK-15 cells (2.0 × 10^5^ cells/well) were pretreated with either ANXA2-specific polyclonal antibody (30 μg/mL) or isotype control antibody (30 μg/mL) at 4 °C for 1 h prior to the addition of PCV2 to the cell culture for 1 h at 4 °C. For a recombinant protein competition assay, PCV2 was preincubated with rANXA2 (20 μg/mL) or BSA (20 μg/mL) at 4 °C for 1 h. The mixture was added to PK-15 cells (2.0 × 10^5^ cells/well) and incubated at 4 °C for another 1 h. After incubation, the cells were washed three times with cold PBS, total DNA was extracted, and the number of PCV2 particles bound to the cells was assessed by detecting ORF2 by qPCR. Each condition was tested in triplicate across at least three independent experiments.

For the immunofluorescence assay, PCV2 VLPs (0.1 μg) were preincubated with rANXA2 (20 μg/mL) or BSA (20 μg/mL) at 4 °C for 1 h and then added to PK-15 cells (0.5 × 10^5^ cells/well) grown on coverslips and incubated at 4 °C for 1 h. After being washed, the cells were fixed with 4% paraformaldehyde without permeabilization, blocked, and incubated with an anti-Cap antibody (stored in our laboratory), followed by an Alexa Fluor 488-conjugated secondary antibody. Nuclei were stained with DAPI. Bound VLPs were observed using a fluorescence microscope, and the average fluorescence intensity was determined (ImageJ/Fiji software).

### Coimmunoprecipitation

293FT cells were seeded in a 6-well plate at a density of 5.0 × 10^5^ cells/well and cotransfected with pCDNA3.4-*ANXA2* (with an HA tag at the C-terminal end) and pCI-neo-PCV2 *cap* (with a Flag tag at the N-terminal end) plasmids using Lipofectamine^TM^2000 (no. 11668019, Invitrogen) for 48 h, after which the cells were lysed in WB/IP buffer and centrifuged at 10 000 × *g* for 5 min. The cleared supernatants were incubated with anti-HA magnetic beads (no. L-1101; Biolinkdin, Shanghai, China) or anti-Flag magnetic beads (no. L-1011; Biolinkdin) overnight at 4 °C with rotation. The samples were eluted with 2 × SDS loading buffer, boiled for 5 min and then subjected to western blot analysis.

### GST pull-down assay

The full-length porcine *ANXA2* gene (FL-*ANXA2*) amplified from porcine lung tissues was subcloned and inserted into the pGEX-4T-1 vector for GST-ANXA2 expression in *E. coli* BL21 cells that were induced with 0.5 mM isopropyl-β-D-1-thiogalactopyranoside (IPTG) at 20 °C overnight. The empty pGEX-4T-1 vector was used to express GST alone under the same conditions. GST-ANXA2 and GST proteins were purified using glutathione resin (Cat. No. L00206; GenScript, Nanjing, China) according to the manufacturer’s instructions. Plasmids encoding Cap with an N-terminal hexa-His tag stored in our laboratory were expressed and purified as described previously [11].

For the pull-down assay, recombinant GST-ANXA2 or GST protein (5 μg) was incubated with anti-GST tag magnetic beads (no. L-2004, Biolinkdin, Shanghai, China) for 2 h at 4 °C. The mixtures were then incubated with purified Cap protein (5 μg) at 4 °C overnight. After incubation, the unbound proteins were removed by washing with PBST. The proteins were subsequently eluted with 2 × SDS loading buffer and subjected to western blot analysis.

### Molecular docking

The interaction and binding sites between ANXA2 and Cap were predicted using AlphaFold3 via the AlphaFold Server. Molecular docking simulations were performed to analyse the interaction interfaces and hydrogen bonding patterns between ANXA2 and Cap. Five top-ranked models were generated, each exhibiting interaction strengths above the predefined threshold. The highest-ranked model was selected and visualized using PyMOL v1.8, with the hydrogen bonds and salt bridges between ANXA2 and Cap shown in this model.

### Statistical analyses

All experiments were repeated at least three times, with consistent results. The data in the figures are the mean ± standard deviation (SD). Statistical analyses included one-way ANOVA and Student’s *t* tests (GraphPad Prism 8.0.2 software, San Diego, CA, USA). Differences between groups were considered significant at *P < 0.05, **P < 0.01, and ***P < 0.001, whereas *P* > 0.05 was deemed a nonsignificant (ns) difference.

## Results

### Identification of ANXA2 as a PCV2 Cap binding protein by LC‒MS/MS

Cap, the primary structural constituent of the PCV2 capsid, plays a crucial role in facilitating viral entry into host cells. To investigate host proteins that interact with PCV2 Cap, PCV2 VLPs were prepared and allowed to bind to PK-15 cells, as previously described [11]. Subsequent identification of potential binding partner(s) was performed with Co-IP and LC‒MS/MS. Finally, several host proteins potentially interacting with PCV2 Cap were identified (Table 1). Among these, ANXA2 was selected for further study because of the high frequency of ANXA2 peptides in LC‒MS/MS analyses, its known roles in membrane repair and cytoskeletal remodelling, and its function as a receptor or coreceptor for other viruses, suggesting its potential involvement during the early stages of PCV2 infection [25, 26]. Although ANXA2 has been previously reported to be associated with infection by other viruses, its role in PCV2 infection has not yet been reported. Therefore, its potential role in PCV2 infection was further investigated.

**Table 1:** **Identification of potential cellular proteins that bind to PCV2-VLPs by LC‒MS/MS in PK-15 cells**

Accession no	Protein	MW (kDa)	Coverage (%)	Unique peptides	PSMs
I3LGD4	Clathrin heavy chain	189.3	24.88	34	39
Q767L7	Tubulin beta chain	49.6	30.18	1	19
A0A287A4R1	Actin beta like 2	41.8	26.33	1	63
A0A287BI04	Annexin A2	36.3	17.08	5	5
A0A287A342	Galectin	33.8	28.15	8	13
I3LUP6	NPM1	32.6	21.77	7	8
A9XFX6	F-actin capping protein beta subunit	30.6	43.75	11	15
A0A286ZK74	hnRNPC	25.3	17.67	4	4
A0A287A7G8	Histone H2B	16.7	36.91	5	6
PSMs: Peptide spectrum matches					

### PCV2 infection induces ANXA2 expression

**Figure 1:**
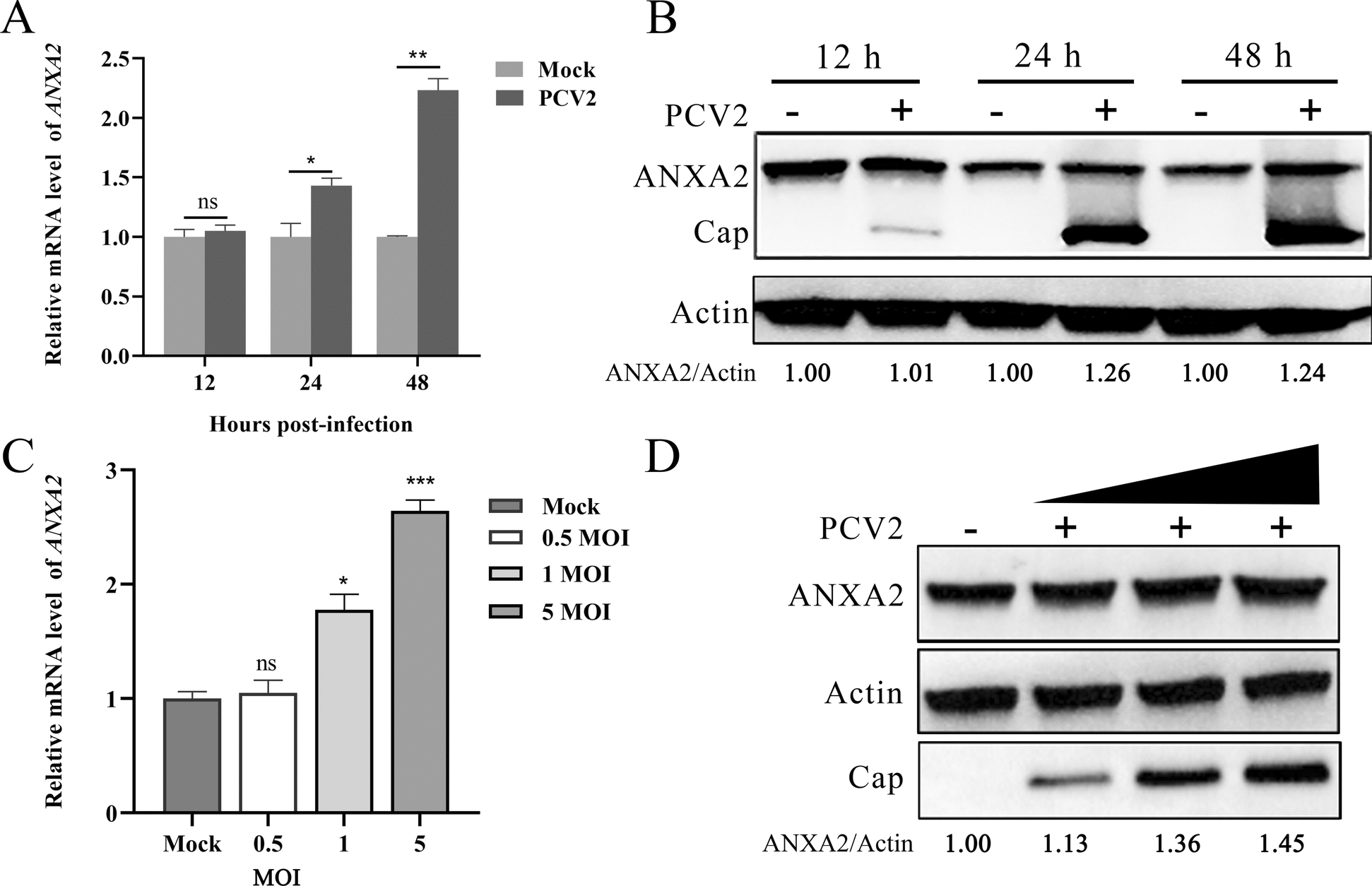
**ANXA2 mRNA and protein expression in PK-15 cells infected with PCV2**.** A**, **B** PK-15 cells were infected with PCV2 at an MOI of 1. Cells were harvested at 12, 24 and 48 hpi, and the mRNA (**A**) and protein (**B**) expression levels of ANXA2 were determined by RT‒qPCR and western blotting. **C**, **D** PK-15 cells were infected with PCV2 at MOIs of 0.5, 1 and 5. At 48 hpi, the mRNA (**C**) and protein (**D**) levels of ANXA2 were determined.

ANXA2 is expressed in various mammalian cells, and its expression can be induced by viral infections or other pathogens [16, 27]. To elucidate the effects of PCV2 infection on ANXA2 expression, PK-15 cells infected with PCV2 were collected and analysed using RT‒qPCR and western blotting. Both ANXA2 mRNA and protein levels were increased at 24 and 48 h post-PCV2 infection (hpi) (Figures 1A, B). Notably, the transcriptional and protein levels of ANXA2 increased in a dose-dependent manner in response to PCV2 infection (Figures 1C, D).

### ANXA2 deficiency inhibits PCV2 proliferation

**Figure 2:**
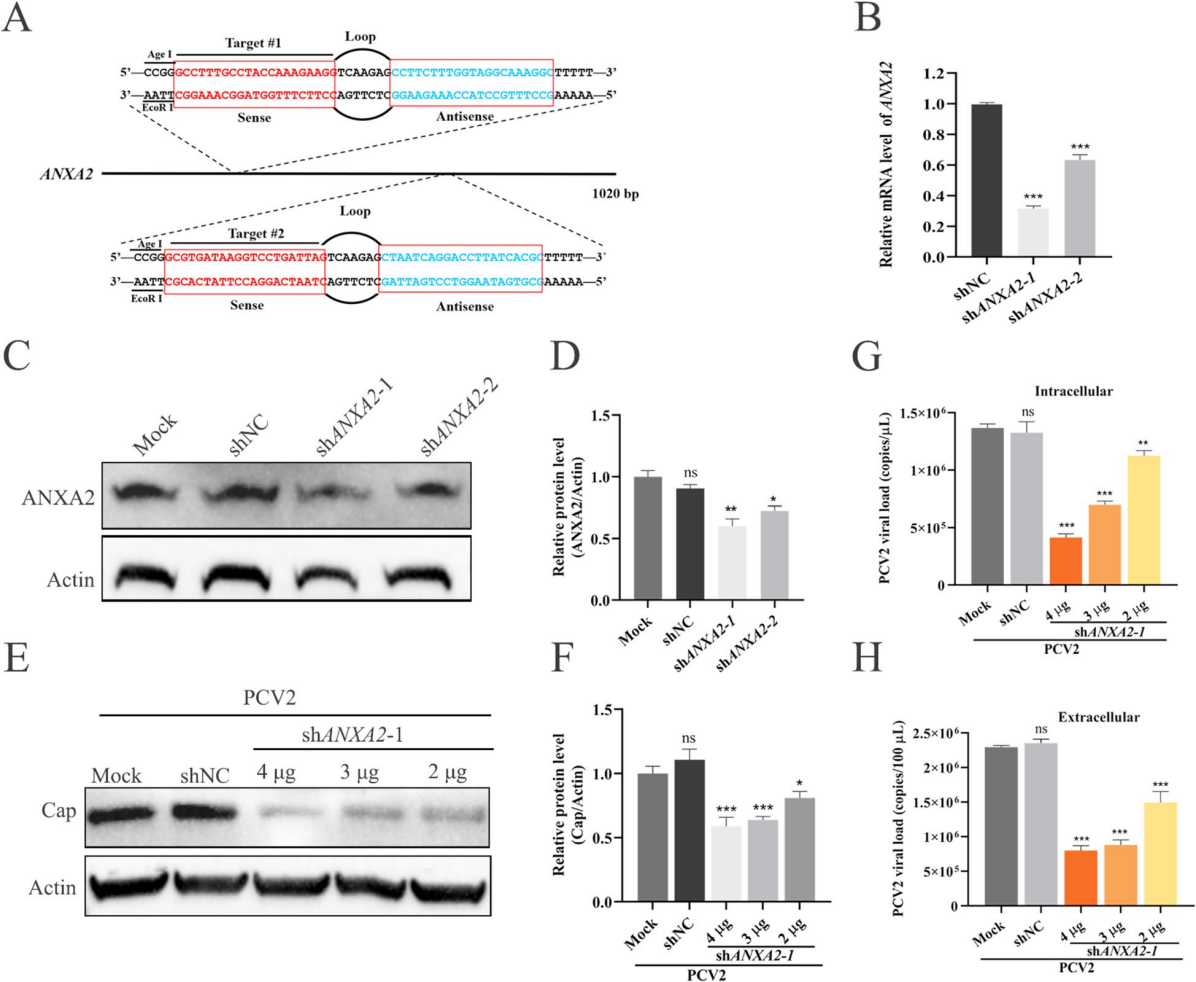
**Effects of ANXA2 knockdown on PCV2 expression in PK-15 cells**.** A** Sequences of specific shRNAs targeting ANXA2 targeted by shRNAs. **B**–**D** Knockdown of endogenous ANXA2. PK-15 cells were transfected with control shRNA or ANXA2 shRNAs; ANXA2 mRNA levels were analysed by RT‒qPCR (**B**), and protein levels were detected by western blotting with an anti-ANXA2 antibody at 24 hpi (**C**, **D**). **E**–**H** Knockdown of ANXA2 inhibited PCV2 replication. PK-15 cells were transfected with 2, 3 or 4 μg of shANXA2-1, 4 μg of shNC or 4 μg of empty vector for 24 h and then incubated with PCV2 for 1 h, after which they were washed with PBS and cultured at 37 °C until 36 hpi. Cells were harvested to detect Cap expression by western blotting with anti-Cap antibody (**E**, **F**), and cellular DNA (**G**) and viral genomic DNA from the supernatant (**H**) were extracted to analyse viral copy numbers by qPCR.

To investigate the influence of ANXA2 on PCV2 proliferation, two specific shRNAs targeting ANXA2 were designed to reduce its mRNA level (Figure 2A). In PK-15 cells, both shRNAs decreased ANXA2 mRNA abundance, with sh*ANXA2*-1 exhibiting a knockdown efficiency of 68.1%, which was much greater than that of sh*ANXA2*-2 (36.4%) (Figure 2B). Similarly, ANXA2 protein levels were reduced by approximately 40.0 and 27.5% in the sh*ANXA2*-1 and sh*ANXA2*-2 groups, respectively (Figures 2C, D). Subsequently, PK-15 cells were transfected with sh*ANXA2*-1 at various doses. Compared with the mock control (transfected with empty vector), sh*ANXA2*-1 dose-dependently decreased the expression levels of PCV2 Cap (Figures 2E, F) and the copy numbers of viral genomic DNA present in cells and extracellular cell cultures (Figures 2G, H). Conversely, compared with the mock-treated cells, the shNC-treated cells did not significantly differ in viral genome copy number. In summary, ANXA2 knockdown significantly reduced PCV2 Cap expression and viral genomic DNA levels, indicating that ANXA2 is critical for efficient PCV2 replication and proliferation.

### A2ti-1 inhibits PCV2 proliferation in vitro

**Figure 3:**
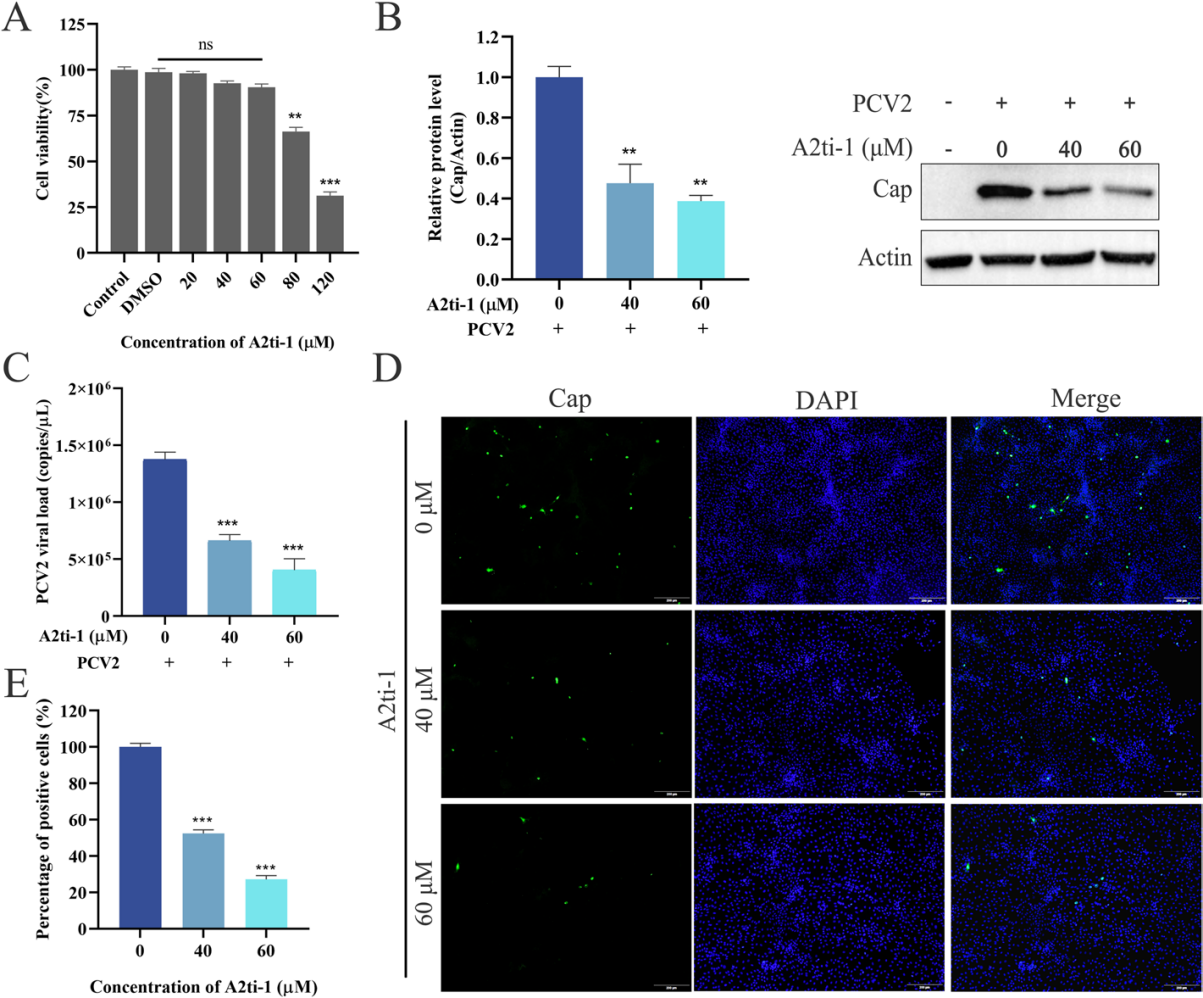
**A2ti-1 suppresses PCV2 proliferation in vitro. A** Cell viability was examined by CCK8 cell viability assays. **B**–**D** A2ti-1 inhibited PCV2 replication in a dose-dependent manner. PK-15 cells were pretreated with the inhibitor A2ti-1 for 2 h and then infected with PCV2 for 1 h. The cells were subsequently washed and further incubated for 36 h, with or without the inhibitor. The expression level of Cap was measured by western blotting (**B**) and IFA (**D**) with an anti-Cap antibody, and viral genome copies were detected by qPCR (**C**). The western blot results were normalized to those of β-actin and quantitated using ImageJ/Fiji software. **E** Percentage of positive cells in Panel D. The percentage of Cap-positive cells was calculated from at least five randomly selected fields. The data represent the results of three independent experiments.

To further investigate the role of ANXA2 in PCV2 infection, A2ti-1, a selective inhibitor of the ANXA2/S100A10 heterotetramer (A2t), was used. Since the A2t complex is essential for the membrane localization and functional activity of ANXA2 in various cellular processes, the inhibition of A2t facilitated the evaluation of the role of membrane-associated ANXA2 in PCV2 replication [28]. Initially, the cytotoxicity of A2ti-1 was assessed using a CCK-8 assay, which revealed a maximum safe concentration of 60 μM in PK-15 cells (Figure 3A). The cells were subsequently pretreated with A2ti-1 at final concentrations of 40 or 60 μM for 2 h prior to PCV2 infection. Western blot analysis revealed that the expression level of Cap decreased in a dose-dependent manner in response to treatment with the A2ti-1 inhibitor; compared with that in the untreated group, the expression of Cap in the group treated with the inhibitor at a concentration of approximately 60 μM was reduced by approximately 60% (Figure 3B). Furthermore, viral genome copy numbers in the cells significantly decreased and reached only 48% and 29% of those in the untreated group when the cells were treated with 40 or 60 μM A2ti-1, respectively (Figure 3C). Similar trends were observed by immunofluorescence assay (IFA) (Figure 3D), wherein a dose-dependent decrease in Cap (green fluorescence) was evident with increasing concentrations of A2ti-1. Taken together, these findings indicate that a specific inhibitor targeting ANXA2 reduced the expression of PCV2 Cap and proliferation of the viral genome in PK-15 cells.

### ANXA2 plays a key role in the early stages of PCV2 infection

**Figure 4:**
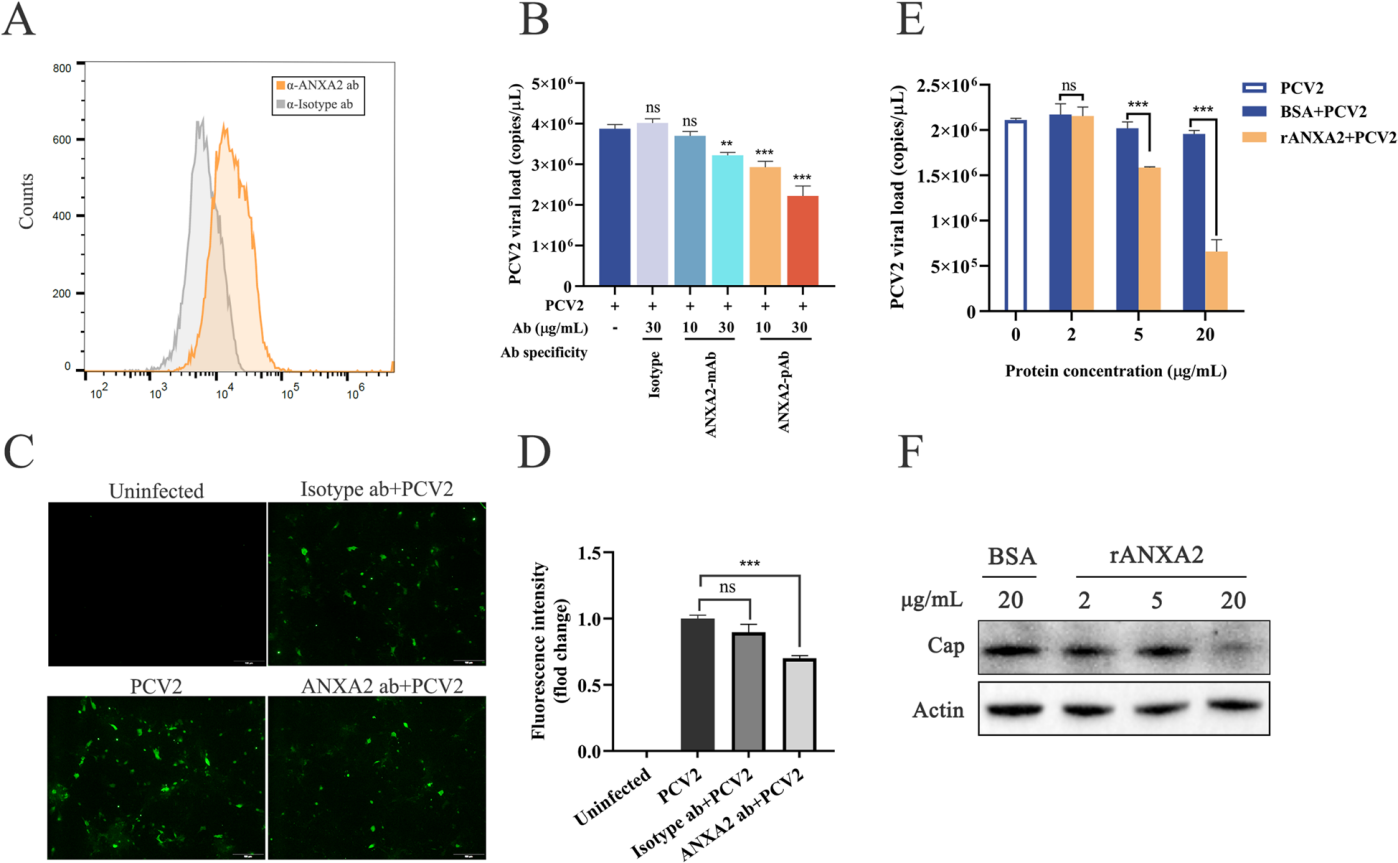
**Cell-surface ANXA2 influences PCV2 infectivity**.** A** Detection of ANXA2 expression on the cell surface of PK-15 cells by flow cytometry. PK-15 cells were incubated with either anti-ANXA2 monoclonal antibody or a rabbit IgG isotype control antibody and then incubated with an Alexa Fluor 488-conjugated donkey anti-rabbit IgG antibody for flow cytometric analysis. **B**–**D** Detecting the effects of ANXA2 on PCV2 infectivity by antibody blocking assays. PK-15 cells were pretreated with anti-ANXA2 mAb, pAb (10 or 30 μg/mL), or rabbit IgG isotype control antibody (30 μg/mL) at 37 °C for 1 h prior to PCV2 infection. The control cells received no antibody. At 36 hpi, viral loads were detected by qPCR (**B**), Cap expression was detected by IFA (**C**), and fluorescence values were measured with ImageJ/Fiji software (**D**). **E**, **F** Infectivity of PCV2 pretreated with rANXA2 in PK-15 cells. PCV2 was preincubated with rANXA2 or BSA (37 °C, 1 h), followed by PK-15 cell infection (1 h). Unbound virus was removed; at 36 hpi, PCV2 DNA was extracted for qPCR (**E**), and Cap protein expression was assessed using western blotting (**F**).

To investigate whether cell surface ANXA2 plays a functional role in early PCV2 infection, antibody blocking assays and exogenous ANXA2 competitive inhibition assays were performed. First, the expression of ANXA2 on the surface of the PK-15 cells was confirmed using flow cytometry, as stronger fluorescence was detected in the cells incubated with the anti-ANXA2 monoclonal antibody than in the control cells, confirming the presence of ANXA2 on the PK-15 cell surface (Figure 4A). To further confirm the membrane localization of ANXA2, PK-15 cells were stained with CellMask™ Green and subjected to nonpermeabilized immunofluorescence staining. Confocal microscopy revealed membrane-associated ANXA2 signals, which was consistent with the flow cytometry results (Additional file 1). Subsequently, PK-15 cells were preincubated with either a rabbit monoclonal antibody recognizing the ANXA2 N-terminal region (amino acids 26–75) or a polyclonal antibody against ANXA2 at concentrations of 10 or 30 μg/mL prior to PCV2 infection, with a rabbit IgG isotype control antibody used as a negative control. Both anti-ANXA2 monoclonal and polyclonal antibodies effectively inhibited PCV2 infection. Specifically, the polyclonal antibody significantly reduced viral genome copies by approximately 24.4% and 42.7% at concentrations of 10 and 30 μg/mL, respectively (Figure 4B), and the monoclonal antibody specifically recognizing the ANXA2 N-terminal region inhibited PCV2 infection by 16.9% at a concentration of 30 μg/mL (Figure 4B). In contrast, no significant change was detected in cells pretreated with the isotype control antibody (Figure 4B). Furthermore, IFA also revealed that PCV2 infectivity was approximately 30% lower in PK-15 cells pretreated with ANXA2-specific polyclonal antibody than in control cells (Figures 4C, D).

To further elucidate the role of surface ANXA2 in PCV2 infectivity, a competitive inhibition assay was conducted with PCV2 pre-incubated with rANXA2 (2, 5 and 20 μg/mL) at 37 °C for 1 h before infection of PK-15 cells. Compared to pre-incubation of PCV2 with BSA before infection, preincubation of PCV2 with rANXA2 resulted in a concentration-dependent reduction of viral genome copy numbers in PK-15 cells (Figure 4E). Moreover, the expression of Cap strongly decreased when PCV2 was incubated with rANXA2 (Figure 4F). These results indicate that interference with PCV2-ANXA2 interactions had a negative effect on PCV2 infectivity, potentially occurring during the early stage of virus‒host binding.

### Surface ANXA2 mediates PCV2 attachment to host cells

**Figure 5:**
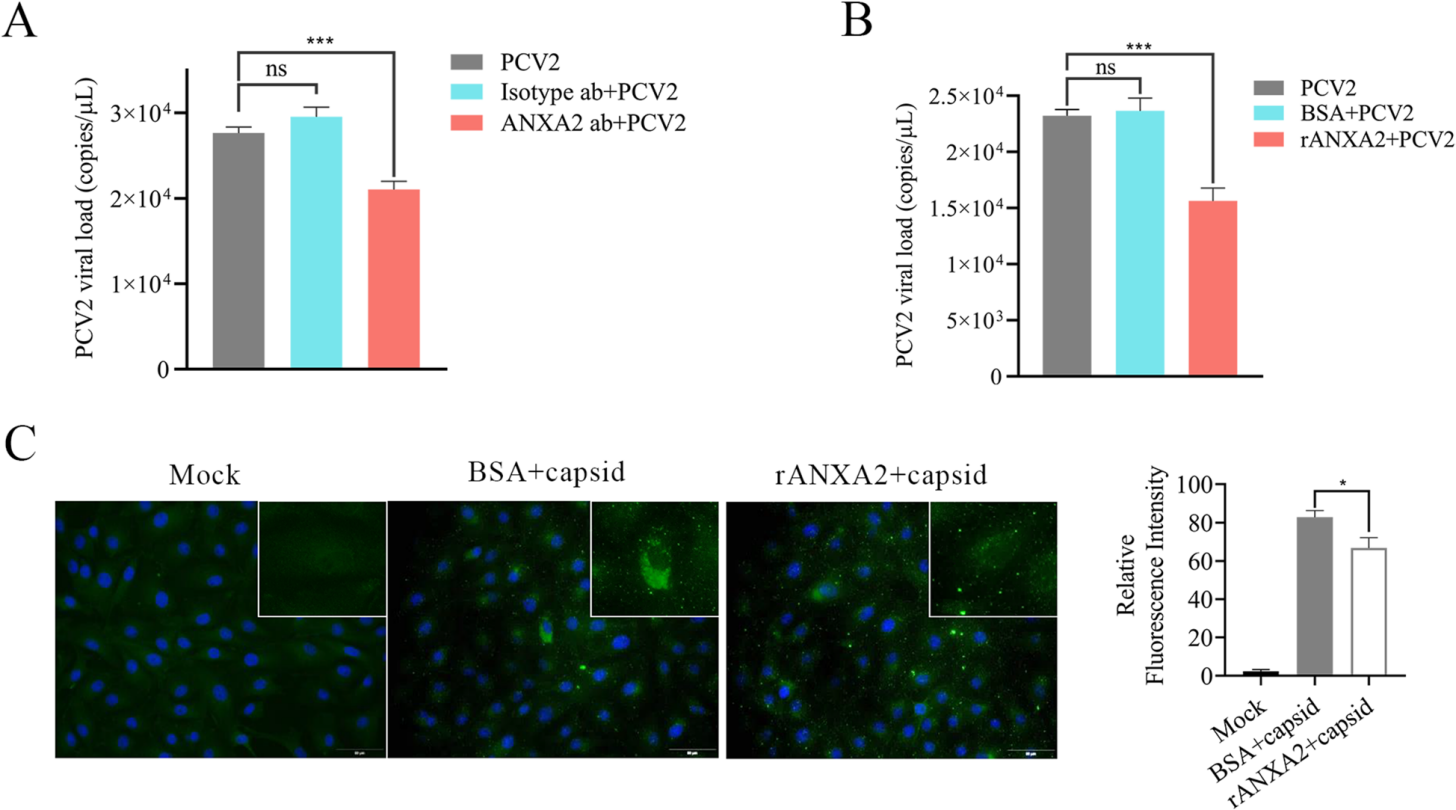
**The role of ANXA2 in the attachment of PCV2 to PK-15 cells**.** A** Pretreatment with ANXA2 antibody and its effects on PCV2 binding to host cells. PK-15 cells were pretreated with ANXA2 antibodies or an isotype control antibody prior to infection with PCV2 at 4 °C for 1 h. The viral DNA copy numbers of the cells were analysed by qPCR. **B** Analysis of PCV2 binding to PK-15 cells using a competitive inhibition assay. PCV2 (MOI of 10) was pretreated with rANXA2 or BSA at 4 °C for 1 h and then incubated with PK-15 cells at 4 °C for 1 h. Bound virus was quantified by qPCR. **C** PCV2 VLPs were mixed with rANXA2 before they were allowed to bind to cells at 4 °C for 1 h. The cells were fixed and then subjected to immunofluorescence detection using an anti-Cap antibody. Bound VLPs were observed using a fluorescence microscope, and the average fluorescence intensity was determined (ImageJ/Fiji software).

Given the role of cell surface ANXA2 in PCV2 infection and its reported role in mediating the attachment of various viruses, additional binding assays were performed to assess the effects of ANXA2 on PCV2 adsorption to host cells. PK-15 cells were pretreated with either ANXA2-specific antibody or isotype control antibody at 4 °C for 1 h prior to the addition of PCV2 to the cell culture, after which the number of PCV2 particles bound to the cells was assessed using qPCR. Compared with no treatment, treatment with ANXA2-specific antibodies reduced the number of attached PCV2 particles by approximately 25.2% (Figure 5A). In contrast, treatment with the isotype had no significant effect on PCV2 attachment to PK-15 cells (Figure 5A). Consistent with the findings from the antibody blocking assay, a competitive binding assay also demonstrated that rANXA2 inhibited PCV2 attachment to cells (approximately 32.7%), whereas BSA had a minimal effect (Figure 5B).

To further confirm the role of cell surface ANXA2 in the binding stage of PCV2, additional experiments were performed using PCV2 VLPs, which are often used in binding assays [9]. The VLPs were preincubated with rANXA2 or BSA prior to binding to PK-15 cells at 4 °C. Bound VLPs were immunolabelled with a specific anti-Cap antibody and visualized by fluorescence microscopy. rANXA2 notably decreased VLP binding to PK-15 cells (approximately 19.4% decrease in fluorescence intensity). In contrast, BSA treatment did not affect the binding of VLPs (Figure 5C). Together, these results demonstrated that cell surface ANXA2 plays a critical role in mediating PCV2 attachment to host cells.

### PCV2 cap interacts with ANXA2

**Figure 6:**
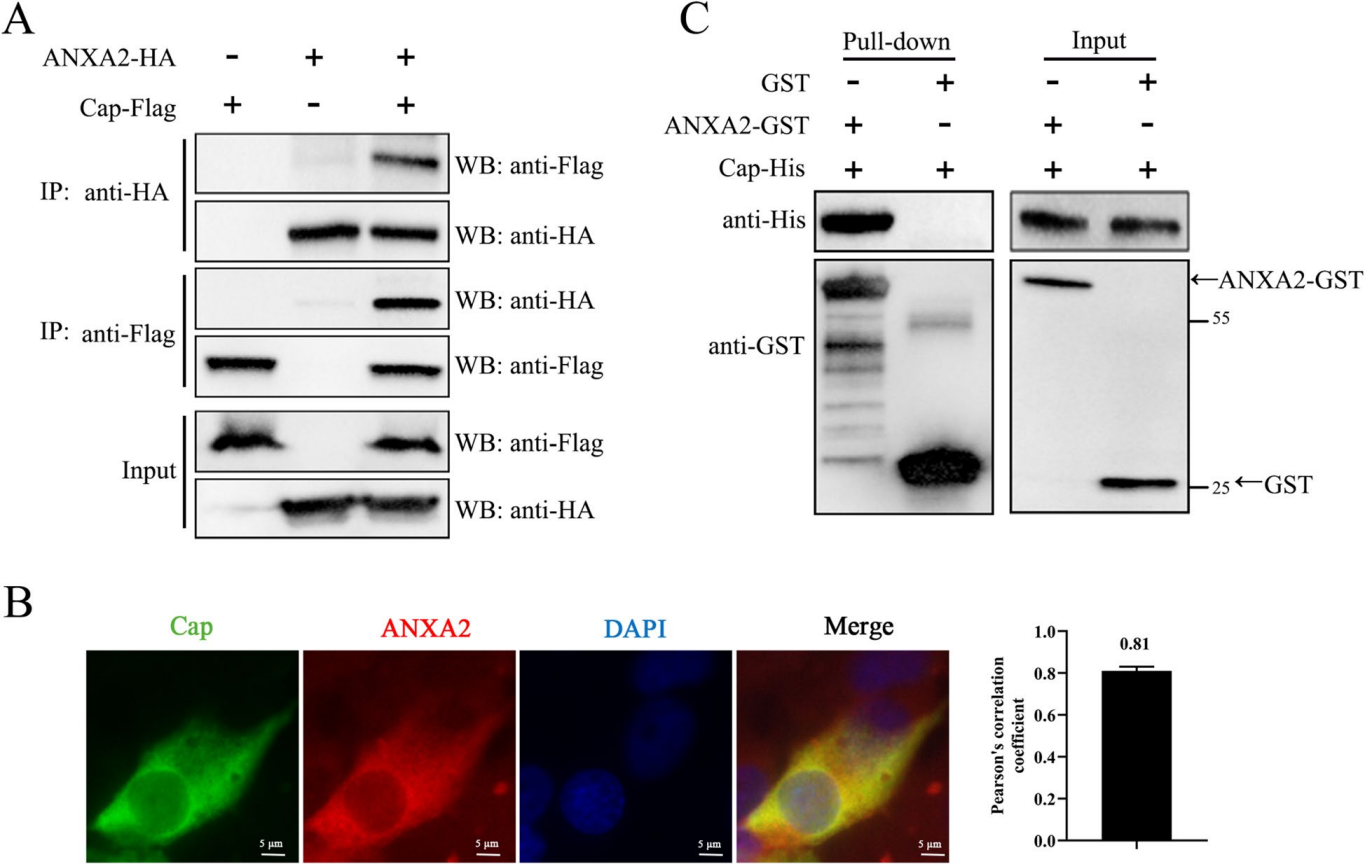
**Interactions between PCV2 Cap and ANXA2**.** A** PCV2 Cap interacted with exogenous ANXA2. 293FT cells were co-transfected with recombinant Cap-Flag and ANXA2-HA and harvested 48 h post-transfection. Immunoprecipitation was conducted with anti-HA or anti-Flag magnetic beads, followed by western blot analysis to identify the eluted proteins. Input lysates were also detected with corresponding antibodies. **B** PCV2 Cap colocalized with endogenous ANXA2. PK-15 cells infected with PCV2 were fixed with 4% paraformaldehyde at 48 hpi, permeabilized with 0.1% Triton X-100, and PCV2 was detected with anti-Cap antibody and stained with Alexa Fluor 488-conjugated goat anti-mouse secondary antibody (green). In addition, ANXA2 was labelled with an anti-ANXA2 antibody and then stained with an Alexa Fluor 594-conjugated goat anti-rabbit secondary antibody (red). The cell nuclei were stained with DAPI (blue). Scale bars = 5 μm. Colocalization was assessed by calculating the Pearson correlation coefficient with ImageJ/Fiji software. **C** Direct interaction between Cap and ANXA2 was determined by a GST pull-down assay. The recombinant GST-ANXA2 protein was bound to anti-GST magnetic beads at 4 °C for 2 h, with the GST protein used as a control. The beads were incubated with His-tagged Cap overnight at 4 °C, after which the samples were eluted and analysed by western blotting with anti-His and anti-GST antibodies.

By identifying ANXA2 as a crucial host protein involved in PCV2 infection, promoting viral entry and enhancing adsorption, the importance of the interaction between Cap and ANXA2 was further confirmed. Co-IP experiments were conducted by co-transfecting plasmids expressing Cap fused with a Flag tag and ANXA2 with an HA tag into 293FT cells. The cell lysates were subjected to immunoprecipitation using antibodies against HA or Flag tags, and the results demonstrated that exogenous ANXA2 interacted with Cap (Figure 6A). Furthermore, confocal microscopy demonstrated the colocalization of endogenous ANXA2 with Cap in PCV2-infected PK-15 cells (Figure 6B), with a Pearson’s correlation coefficient of 0.81, suggesting that an interaction occurred.

Binding assays demonstrated that ANXA2 facilitated viral adsorption to host cells (Figure 5). Given that Cap plays a role in virus entry through interactions with host proteins, we hypothesized that there might be a direct interaction between them. GST pull-down assays confirmed that Cap bound directly to GST-ANXA2 but not to the control GST alone (Figure 6C). These results collectively confirmed that Cap directly interacted with ANXA2 in vitro.

### Identification of the binding domains of ANXA2 that interact with cap

**Figure 7:**
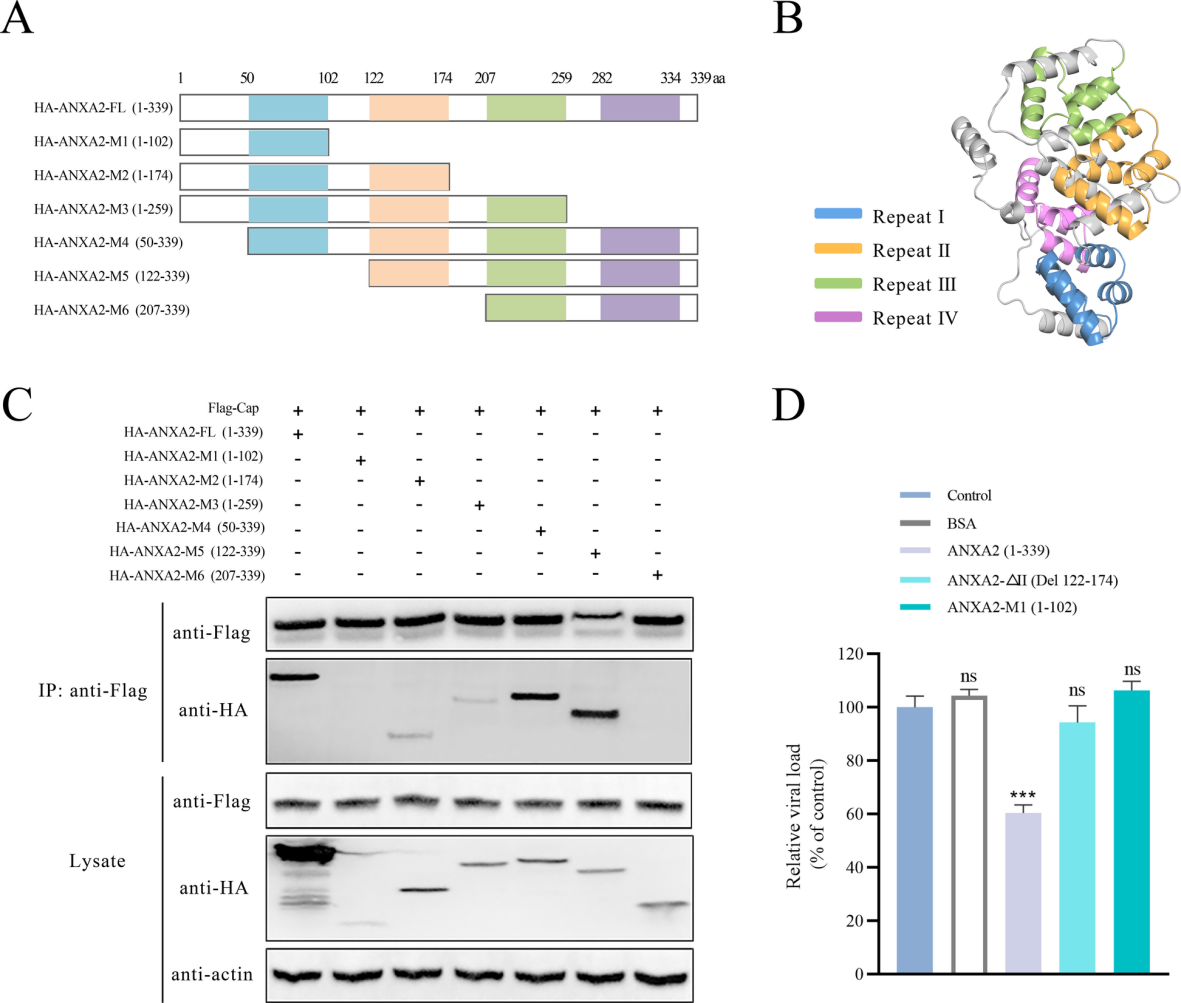
**Identification of the key domains of ANXA2 that interact with Cap in 293FT cells**.** A** Schematic mapping of ANXA2 domains and its truncation mutants. FL-ANXA2 and six ANXA2 truncation mutants tagged with HA were constructed. **B** Structural model of *Sus scrofa* ANXA2 generated by AlphaFold 3 and coloured by repeats. The blue represents repeat I (50–102 aa), the orange represents repeat II (122–174 aa), the green represents repeat III (207–259 aa) and the purple represents repeat IV (282–334 aa). **C** Cap was co-transfected with either FL-ANXA2 or its various truncates into 293FT cells; these cells were then subjected to Co-IP with an anti-Flag antibody and subsequently analysed by western blotting with the indicated antibodies. **D** Effects of FL-ANXA2, ANXA2-△II and ANXA2-M1 on PCV2 attachment to PK-15 cells. PCV2 was preincubated with various proteins (expressed in *E. coli* and used at equal concentrations) at 37 °C for 1 h, followed by infection of PK-15 cells at 4 °C for 1 h. Bound virus was quantified by qPCR.

ANXA2 consists of two main structural domains, the N-terminal domain and the C-terminal core domain, with the latter containing four highly conserved repeating structures (I–IV) [29]. To determine which domain(s) of ANXA2 was crucial for its interaction with Cap, six HA-tagged ANXA2 truncation mutants were constructed and co-expressed with Flag-tagged Cap in 293FT cells. Co-IP experiments revealed that Cap interacted with full-length (FL) ANXA2 and that all the proteins were truncated except the M1 and M6 mutants (Figure 7C), indicating that repeat II (122–174 aa) in the C-terminal core domain of ANXA2 may be responsible for interactions between the two proteins.

To further evaluate the functional relevance of ANXA2 repeat II (122–174 aa) in PCV2 attachment, expression of the isolated repeat II fragment in *E. coli* was attempted but was not successful (data not shown). As an alternative approach, a deletion mutant lacking repeat II (ANXA2-△II) was constructed. In addition, the ANXA2-M1 mutant (1–102 aa) and BSA were included as negative controls. Full-length ANXA2 (FL-ANXA2), ANXA2-△II, and ANXA2-M1 were expressed and purified from *E. coli*. PCV2 was preincubated with each protein at an equal concentration of 20 μg/mL at 37 °C and then incubated with PK-15 cells at 4 °C for 1 h. As shown in Figure 7D, FL-ANXA2 significantly inhibited PCV2 attachment, whereas neither ANXA2-△II nor ANXA2-M1 (1–102 aa) nor BSA had any significant effect. Thus, these results demonstrate that repeat II (aa 122–174) of ANXA2 not only mediates its interaction with Cap but also plays a key role in facilitating PCV2 attachment.

### Molecular docking of cap to ANXA2

**Figure 8:**
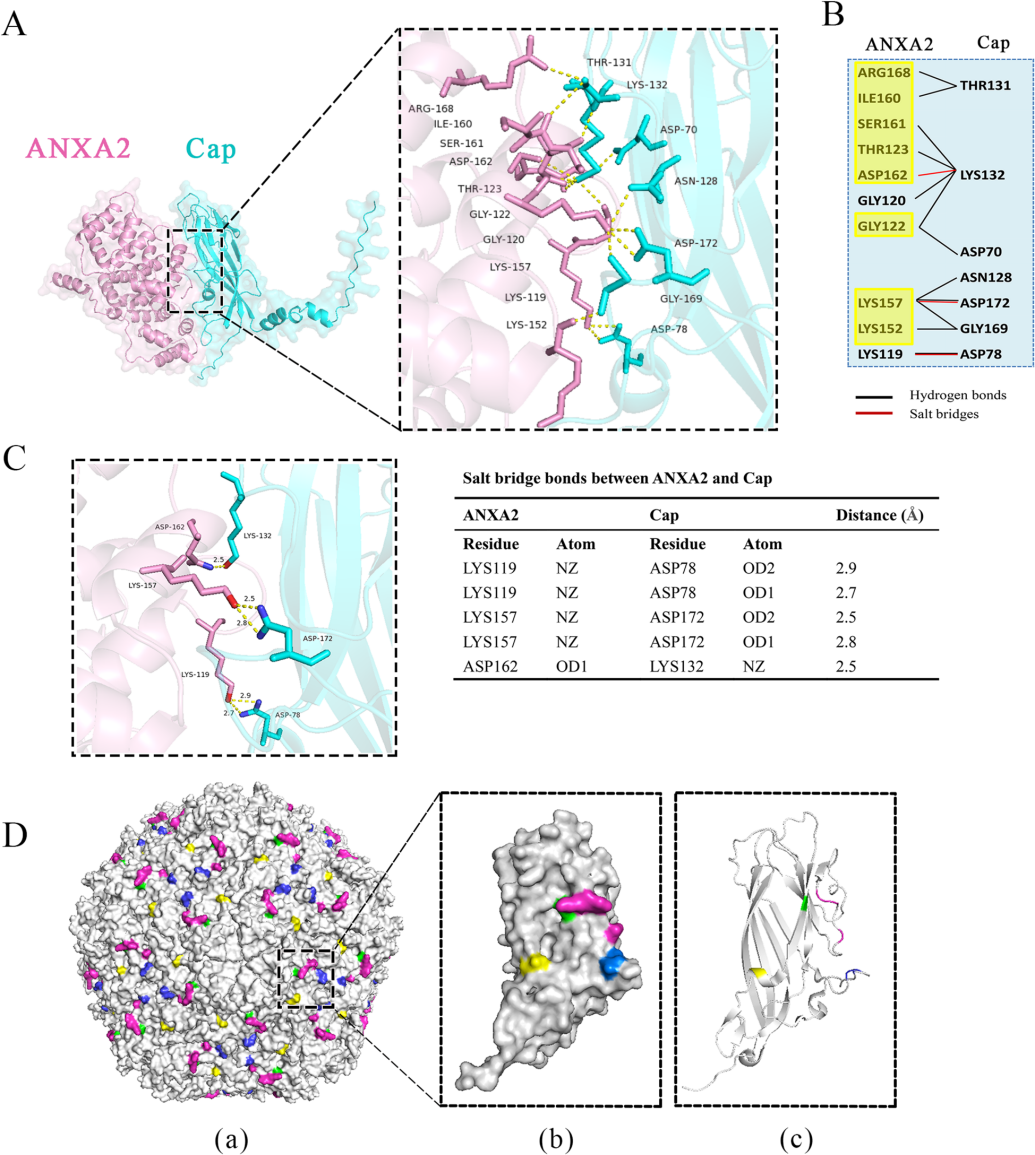
**Structural modelling of Cap and ANXA2**. Molecular docking of ANXA2 (pink) and Cap (cyan) was predicted using AlphaFold3. Interacting interfaces are highlighted within dashed boxes. Zoomed-in views show key amino acid residues at the interface, with hydrogen bonds and salt bridges shown in yellow. **B** List of amino acid residues at the interface. The black and red lines represent hydrogen bonds and salt bridges, respectively. Amino acids located in ANXA2 repeat II are marked in yellow. **C** Salt bridge interactions at the interface between ANXA2 and Cap. **D** 3D structure of the interacting residues in the loops of Cap and their distributions on the PCV2 VLP surface are labelled with distinct colours. Violet represents Loop EF (Asn128, Thr131 and Lys132), blue represents Loop GH (Gly169 and Asp172), yellow represents Loop CD (Asp78), and green represents the β-sheet (Asp70) on the PCV2 Cap. **Panel (a)**: surface view of the PCV2 VLP (capsid); **Panel (b)**: surface view of the PCV2 Cap subunit; and **Panel (c)**: crystal structure of the PCV2 Cap.

To elucidate the molecular mechanism through which ANXA2 interacts with Cap, AlphaFold3 was used to simulate interactions between *Sus scrofa* ANXA2 and Cap. After molecular docking, the predicted template modelling (pTM) score, a confidence metric generated by AlphaFold3, was > 0.5, indicating that the predicted fold of the protein complex closely resembled the true biological structure [30]. On the basis of the per-residue confidence scores (pLDDTs) and the p™ score (0.62) of the predicted models, higher accuracy and confidence were demonstrated in the structure shown in Additional file 2. Residues involved in salt bridges and hydrogen bonds were identified as key contributors to binding affinity. Docking analysis revealed multiple hydrogen bonds and salt bridges at the interface between Cap and ANXA2 (Figure 8A). These interactions were attributed primarily to residues in loop regions of Cap (Asp78 in Loop CD; Asn128, Thr131 and Lys132 in Loop EF; and Gly169 and Asp172 in Loop GH), together with Asp70 located on a β-sheet of Cap and ANXA2 repeat II (amino acids 122–174) (Figures 8A, B). The interface was further analysed at 3 Å; three salt bridges between ANXA2 and Cap (Lys119-Asp78, Lys157-Asp172, and Asp162-Lys132) dominated the interaction (Figure 8C). Since PCV2 Cap interacts with ANXA2 through capsid/VLPs assembled from 60 of the Cap subunits, residues exposed on the surface of the Cap subunit may not have been present on the capsid surface again after assembly. Thus, we labelled these residues onto the Cap assembly PCV2 VLPs (PDB: 3R0R) (Figure 8D). Furthermore, all the interacting residues of Cap were also exposed on the capsid surface, providing strong evidence that molecular docking may represent the true biological process at the early stage of viral infection. Taken together, these findings underscore the critical role of repeat II of ANXA2 in mediating the interaction between ANXA2 and Cap and suggest that ANXA2 may bind directly to amino acids exposed on the surface of viral particles.

## Discussion

PCV2 relies on host cells to complete its life cycle; therefore, investigating virus‒host interactions is crucial for understanding viral infection mechanisms and formulating treatment strategies. Research on PCV2 receptors and other potential molecules involved during early infection is relatively limited. Although GAGs have been identified as receptors for PCV2, the ability of PCV2 to infect Chinese hamster ovary (CHO) cells lacking GAG synthesis suggests that unidentified cellular factors may participate in viral adsorption [10]. In this study, PCV2 VLPs were used to investigate cellular entry in PK-15 cells. ANXA2 was identified (by immunoprecipitation and LC‒MS/MS) as a novel host protein that interacts with Cap. Furthermore, ANXA2 was confirmed to serve as an accessory factor facilitating PCV2 adsorption. These findings identify ANXA2 as a new host protein that interacts with PCV2 Cap and highlight its role in PCV2 attachment, deepening the understanding of PCV2 infection mechanisms.

Cap is the sole structural protein of PCV2, and 60 of the subunits can self-assemble into a functional capsid in vivo. Thus, it is crucial to elucidate the molecular mechanisms by which PCV2 interacts with host cells via the Cap/capsid and various cellular factors during infection and to develop effective clinical strategies for PCV2 control. Immunoprecipitation combined with LC‒MS/MS is very effective for identifying PCV2 receptors [31]. In this study, rather than PCV2 virions or expressing *cap* gene, PCV2 VLPs were used to screen Cap-specific host factors implicated in cellular entry of the virus. Using VLPs avoided interference from viral nucleic acids or nonstructural viral components associated with productive infections [32]. Additionally, conventional transfection protocols are restricted by prolonged incubation and inefficient Cap protein yield, affecting the detection of transient interactions. These limitations were addressed by using VLPs, which maintain native protein conformations without exogenous expression systems. Moreover, VLPs exhibit surface structures very similar to their viral counterparts, which may mimic the process of cellular entry of viruses, making VLPs an ideal model for studying interactions between PCV2 and host factors [33]. Using this strategy, we successfully identified multiple host proteins that potentially interact with Cap, among which ANXA2, a multifunctional host protein, was selected as a candidate in this study.

The levels of both ANXA2 mRNA and protein were upregulated during PCV2 infection, which is consistent with the findings of previous studies on other viral infections [34], bacterial invasion [16], and cancer development [35]. Given the ubiquitous expression of ANXA2 in mammalian cells and its substantial homologues across species, ANXA2 may play important roles in viral infections [36]. Several studies have demonstrated that knocking down ANXA2 in cells inhibited the replication of several viruses [37–39]. Similarly, in this study, knocking down ANXA2 in PK-15 cells significantly reduced the expression of PCV2 Cap and the number of intracellular and extracellular PCV2 genome copies (Figure 2), demonstrating that ANXA2 promoted PCV2 replication. Moreover, the specific inhibitor A2ti-1, which targets ANXA2/S100A10, exhibited antiviral effects against PCV2, which is consistent with its effects against other viruses (e.g., HPV16, PRV, and ARV [18, 20, 39]). Specifically, A2ti-1 significantly reduced the expression of Cap and viral genome replication in PK-15 cells, emphasizing the critical role of ANXA2 in PCV2 replication. A2ti-1 specifically and efficiently disrupted the formation of the ANXA2/S100A10 complex. Notably, previous studies have demonstrated that S100A10 is critical for the localization of ANXA2 on the cell membrane [40, 41]. The finding that A2ti-1 inhibited PCV2 infection in PK15 cells suggests that the location of ANXA2 on the cell membrane is a key step in facilitating PCV2 entry into host cells for infection. Thus, A2ti-1 has potential as a drug candidate for blocking PCV2 infection, and further in vivo studies are needed to evaluate whether A2ti-1 or its analogues could be used clinically to target PCV2.

The host factor ANXA2 is essential for the life cycles of various viruses, including HPV, human cytomegalovirus (HCMV), hepatitis B virus (HBV), EV71, and respiratory syncytial virus (RSV) [36, 42]. Surface membrane-associated ANXA2 facilitates viral and bacterial adhesion, particularly during cell attachment and entry, as previously reported [25, 43]. For instance, ANXA2 interacts with the LppA surface adhesion protein of *Mycoplasma bovis*, promoting bacterial adherence to host cells [16, 44]. Similarly, ANXA2 binds to VP1, a primary structural protein constituting EV71 particles, enhancing viral infectivity by facilitating viral attachment, highlighting its function as an accessory factor during viral entry [45]. Prior to investigating the role of membrane-surface ANXA2 in PCV2 infection, we first confirmed its expression on the surface of PK-15 cells by flow cytometry, which was consistent with previous findings that demonstrated the surface localization of ANXA2 in RD cells using the same technique [45]. Antibodies targeting ANXA2 blocked HIV-1 infection of macrophages or EV71 infection of RD cells [45, 46]. Similarly, specific antibodies against ANXA2 dose-dependently blocked PCV2 infection (Figure 4B). Notably, at equivalent concentrations, polyclonal antibodies targeting ANXA2 had stronger inhibitory effects than monoclonal antibodies targeting its N-terminus did (Figure 4B), indicating that the C-terminus of ANXA2 may play a pivotal role in PCV2 infection. Considering the ability of the ANXA2 C-terminus to bind to HSPGs, its interaction with these molecules may further stabilize virus particles on the cell surface, thereby promoting the subsequent internalization of the virus [17, 18]. Additionally, its interaction with F-actin enhances the stability of the actin cytoskeleton [47–49]. As PCV2 attachment and internalization depend on HSPGs and F-actin, perhaps the C-terminal domain of ANXA2 influences PCV2 internalization through interactions with HSPGs or F-actin [12, 50]. Overall, we propose that ANXA2 and HSPGs (or HS-containing GAGs) may function in a cooperative or sequential manner during PCV2 infection. ANXA2 may depend on HSPG-associated HS for membrane localization or interact with PCV2 in HS-enriched microdomains. Although viral attachment was only partially inhibited by blocking ANXA2, the observed reduction highlights its auxiliary role (Figure 5). Further investigations into ANXA2 interactions with membrane components, especially GAGs, in PCV2 infection are needed.

Adsorption is the first step of infection, and whether host proteins involved in the early stages of PCV2 infection participate in this process is unclear. The PCV2 capsid, assembled by 60 Cap subunits, mediates interactions with cell receptors. Consequently, cell surface proteins (e.g., ANXA2) that interact with Cap are likely involved in binding PCV2 to host cells. Therefore, we further investigated the role of ANXA2 on the surface of PK-15 cells in the binding process of PCV2. Both qPCR and IFA supported our hypothesis, confirming that exogenous ANXA2 protein or ANXA2-specific antibodies significantly inhibited PCV2 binding (Figure 5). Additionally, the interaction between Cap and ANXA2 was verified in 293FT cells by co-IP (Figure 6A), and the direct interaction of these two proteins was further confirmed by GST-pull down assays (Figure 6B). These findings validated the critical role of ANXA2 in promoting PCV2 attachment to host cells.

Previous studies have highlighted the importance of the C-terminal region (268–334 aa) of ANXA2 in the replication of bovine ephemeral fever virus [51]. Other studies have emphasized the role of full-length ANXA2 in the replication of porcine reproductive and respiratory syndrome virus (PRRSV), whereas the 3D polymerase of EV71 interacts with any domain of ANXA2, and the N-terminal domain is not essential for this interaction [38, 52]. In contrast, our experiments demonstrated that ANXA2 repeat II (122–174 aa) was essential for its interaction with the Cap protein. These differences likely reflected distinct mechanisms of ANXA2 in viral infections. To further investigate the functional role of repeat II (122–174 aa), we attempted to express this domain alone in *E. coli* but failed (data not shown), possibly because of its small molecular weight or instability in the absence of its native structural context. We therefore constructed a deletion mutant lacking repeat II (ANXA2-△II). Preincubation of PCV2 with ANXA2-△II failed to block viral attachment, in contrast to full-length ANXA2, indicating that repeat II is not only necessary for Cap binding but also functionally required for ANXA2-mediated inhibition of viral attachment. Molecular docking using AlphaFold3 confirmed that ANXA2 repeat II formed multiple hydrogen bonds with Cap residues, emphasizing its critical role in this interaction. The Ca^2^⁺- and membrane-binding sites are distributed mainly on the convex face of ANXA2 [25]. In our study, the ANXA2 region (122–174 aa) interacts with Cap and spans a relatively large and flexible portion of its core domain, which makes it unsuitable for strict assignment to either convex or concave surfaces. Nevertheless, considering the conformational flexibility of ANXA2 during membrane association [40], this region may remain accessible for protein interactions, even in the membrane-bound state. Importantly, Cap residues are located on the surface of the PCV2 capsid, suggesting a direct interaction between Cap and ANXA2 during initial virus attachment. Thus, these findings validate the critical role of ANXA2 in promoting PCV2 attachment to host cells.

In conclusion, ANXA2 was identified as a cellular adhesion factor that facilitates PCV2 attachment. Furthermore, ANXA2 directly interacted with Cap, promoting PCV2 binding to host cells and increasing viral infectivity. These insights expand the understanding of PCV2 infection mechanisms and provide new information for the development of antiviral strategies against PCV2.

## Supplementary Information

**Additional file 1** **Surface localization of ANXA2 in PK-15 cells.** PK-15 cells were stained with CellMask™ Green plasma membrane dye (Invitrogen) and immunostained for ANXA2 under nonpermeabilized conditions. Confocal microscopy revealed membrane-associated ANXA2 signals, which was consistent with the flow cytometry results. Scale bar: 50 μm.

**Additional file 2** **Model of the ANXA2-Cap complex coloured by model confidence (pLDDT).** p^TM^, predicted template modelling score. ip^TM^, interface-predicted template modelling score. Right panel: Expected position error plot.

## Data Availability

The data that support the findings of this study are available on request from the corresponding author.
