# Supplementary figures and images for: Annexin A2 facilitates porcine circovirus type 2 infection by mediating viral attachment to host cells and interacting with the capsid protein
Source: Vet Res. 2025 Oct 10;56:191. doi: 10.1186/s13567-025-01628-3 (PMC12512739; doi:10.1186/s13567-025-01628-3)

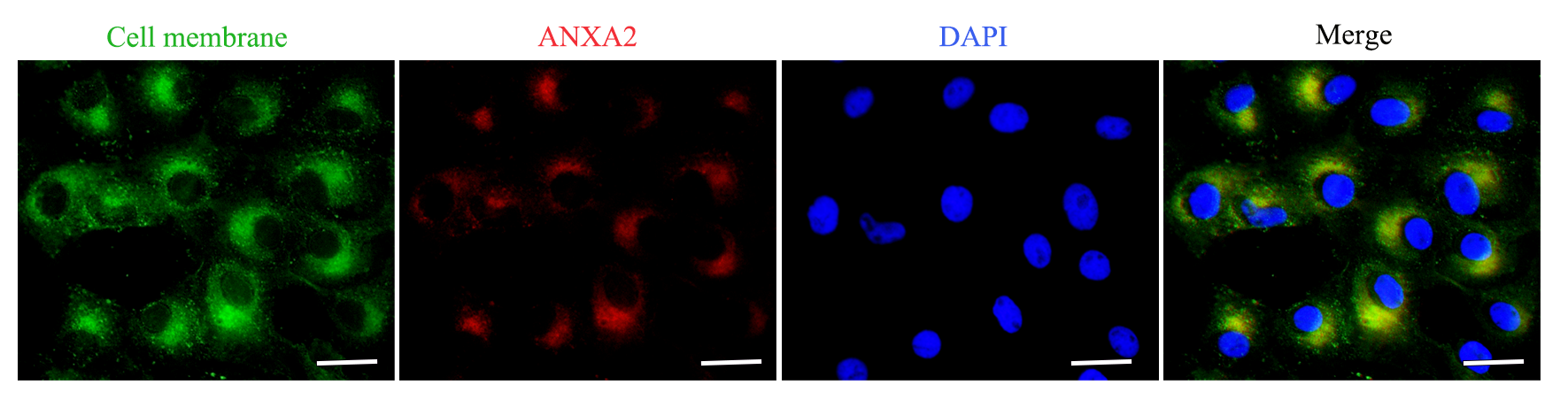

Supplement: Supplementary file 1 — Additional file 1 Surface localization of ANXA2 in PK-15 cells. PK-15 cells were stained with CellMask™ Green plasma membrane dye (Invitrogen) and immunostained for ANXA2 under nonpermeabilized conditions. Confocal microscopy revealed membrane-associated ANXA2 signals, which was consistent with the flow cytometry results. Scale bar: 50 μm. [file 13567_2025_1628_MOESM1_ESM.png]

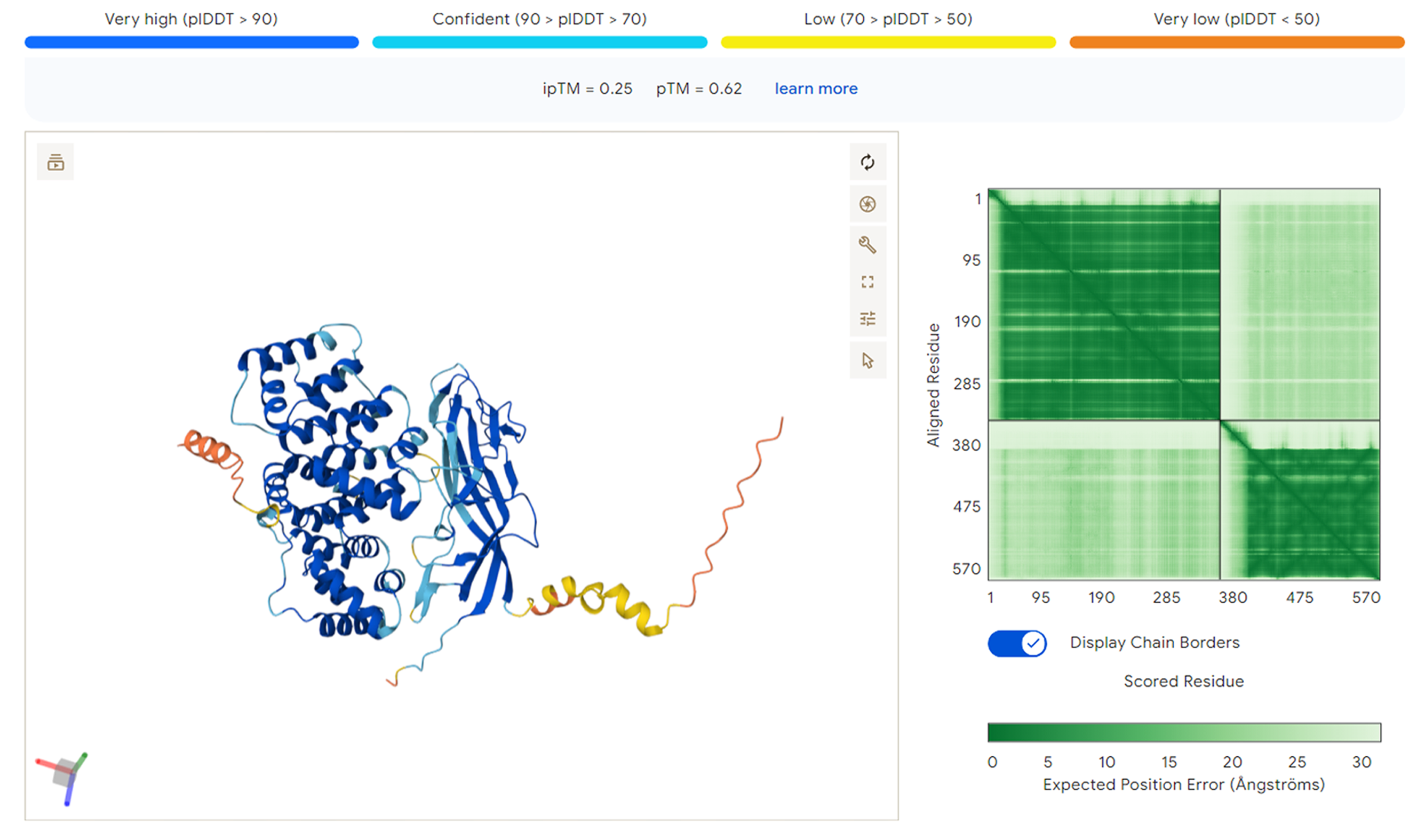

Supplement: Supplementary file 2 — Additional file 2 Model of the ANXA2-Cap complex coloured by model confidence (pLDDT). pTM, predicted template modelling score. ipTM, interface-predicted template modelling score. Right panel: Expected position error plot. [file 13567_2025_1628_MOESM2_ESM.png]
